# Controllable synthesis of mesostructures from TiO_2_ hollow to porous nanospheres with superior rate performance for lithium ion batteries†
†Electronic supplementary information (ESI) available: Experimental section, thermogravimetric heating curves, SEM micrographs, additional TEM micrographs, Raman spectra, N_2_ adsorption–desorption results, EIS after 3 cycles at a current of 1 C and a summary of the cycling performance at various charge/discharge current densities are included. See DOI: 10.1039/c5sc03203b



**DOI:** 10.1039/c5sc03203b

**Published:** 2015-10-26

**Authors:** Hao Ren, Jiajia Sun, Ranbo Yu, Mei Yang, Lin Gu, Porun Liu, Huijun Zhao, David Kisailus, Dan Wang

**Affiliations:** a Department of Physical Chemistry , School of Metallurgical and Ecological Engineering , University of Science & Technology Beijing , No. 30, Xueyuan Road, Haidian District , Beijing 100083 , P. R. China . Email: ranboyu@ustb.edu.cn; b National Key Laboratory of Biochemical Engineering , Institute of Process Engineering , Chinese Academy of Sciences , No. 1, Bei Er Tiao, Zhongguancun , Beijing 100190 , P. R. China; c Laboratory for Advanced Materials & Electron Microscopy , Beijing National Laboratory for Condensed Matter Physics , Institute of Physics , Chinese Academy of Sciences , Beijing 100190 , P. R. China; d Centre for Clean Environment and Energy , Gold Coast Campus , Griffith University , Southport , Queensland 4222 , Australia; e Department of Chemical and Environmental Engineering , University of California , Riverside , CA 92521 , USA

## Abstract

Uniform TiO_2_ nanospheres from hollow, core–shell and mesoporous structures have been synthesized using quasi-nano-sized carbonaceous spheres as templates. The TiO_2_ nanospheres formed after calcination at 400 °C are composed of ∼7 nm nanoparticles and the shells of the hollow TiO_2_ nanospheres are as thin as a single layer of nanoparticles. The ultrafine nanoparticles endow the hollow and mesoporous TiO_2_ nanospheres with short lithium ion diffusion paths leading to high discharge specific capacities of 211.9 and 196.0 mA h g^–1^ at a current rate of 1 C (167.5 mA g^–1^) after 100 cycles, and especially superior discharge specific capacities of 125.9 and 113.4 mA h g^–1^ at a high current rate of up to 20 C. The hollow and mesoporous TiO_2_ nanospheres also show superior cycling stability with long-term discharge capacities of 103.0 and 110.2 mA h g^–1^, respectively, even after 3000 cycles at a current rate of 20 C.

## Abstract 2

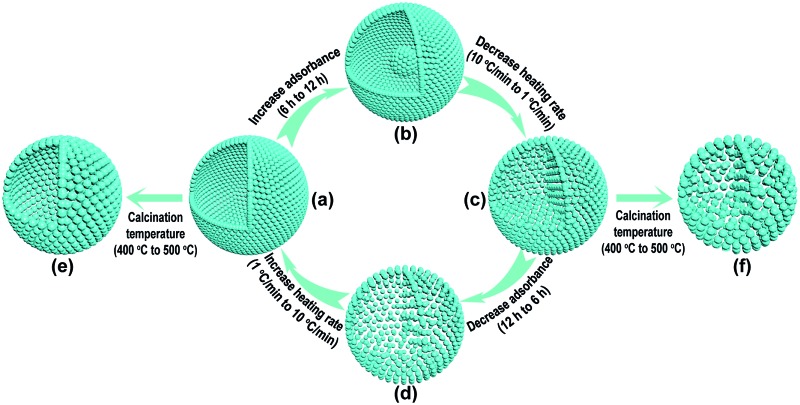
Uniform TiO_2_ nanospheres formed from hollow and mesoporous nanospheres composed of 7 nm sized nanoparticles have been synthesized and show superior rate performance as anode materials for lithium ion batteries.

## Introduction

Lithium-ion batteries (LIBs) are one of the most promising and widely used technologies because of their safety, stable cycle life and low cost.^1^ Due to the great demand for high capacity LIBs, potential candidates have been intensively studied for decades.^2–5^ Recently, transition metal oxides such as Fe_2_O_3_, Co_3_O_4_, Mn_2_O_3_, CuO and NiO have been introduced as anodes and show higher capacities.^6–14^ However, since these anode materials are used at low discharge voltages (below 1 V *vs.* Li^+^/Li), they suffer from the formation of an unstable solid electrolyte interface (SEI) film on the electrode surface, which affects their safety.^15,16^ Recently, with the blossom of electric vehicles, highly powered and safer anodes are necessary.^17^ In view of this, anode materials like Li_4_Ti_5_O_12_ and TiO_2_ with high discharge voltages under which the electrolyte is stable are of great interest.^18–20^

TiO_2_ is widely applied in many fields including environmental remediation, sensing and energy applications.^21–24^ As an anode material for LIBs, it has a high discharge voltage (up to 1 V) which can prevent the formation of a SEI film. In addition, the volume expansion during lithiation and delithiation processes is ∼3%, which reduces strain and permits a stable morphology resulting in a long cycle life.^25^ Moreover, TiO_2_ is abundant, environmentally friendly and chemically stable. However, the limited specific capacity and poor cycling performance of TiO_2_ resulting from the low ionic and electrical conductivities have obstructed its practical use.^26–28^ An effective way to decrease the lithium ion diffusion path lengths is to use nanosized TiO_2_ particles. Thus, the mean diffusion time of the lithium ions in electrode materials could be strongly decreased in nanoparticles according to the following formula: *T*_eq_ = *L* ^2^/*D* (*T*_eq_: mean diffusion time, *L*: diffusion length, *D*: diffusion coefficient).^29,30^ Unfortunately, in practical applications, the aggregation of nanoparticles during the charge/discharge processes will lead to another challenge.^31^ In order to solve these problems, purposely designed hierarchical mesostructures such as hierarchical microspheres, hollow microspheres and core–shell hollow microspheres have been proposed and synthesized. These micro-sized particles can provide good structural stability while nanoparticles can enhance the interfacial contact area and shorten the transition path of the lithium ions and electrons.^32–35^ We have successfully designed and fabricated mesostructured multi-shelled Fe_2_O_3_ and TiO_2_ hollow spheres which can improve the specific capacity, and have demonstrated that thin shelled microspheres show a better performance compared to thick shelled spheres.^7,36^ To our knowledge, no reports focus on very thin shelled hollow spheres. Moreover, the nanoparticles of the multi-shelled TiO_2_ hollow microspheres are currently larger than 20 nm.^36^ We anticipate that smaller sized nanoparticles are more favorable for shorter lithium ion diffusion times, thus leading to a better rate performance.

Herein we have successfully prepared TiO_2_ hollow nanospheres (TiO_2_-HNSs) with much thinner shells composed of nanoparticles as small as about 7 nm using a quasi-nano-sized carbonaceous sphere template method. The shell of the TiO_2_-HNSs was found to be as thin as a single layer of nanoparticles. By tuning the precursor absorbance and calcination processes, TiO_2_ mesoporous nanospheres (TiO_2_-PNSs) have also been fabricated. These kinds of nanospheres show superior performance, especially at high charge/discharge current rates. This results from their hollow and porous structures, which can provide easy charge transfer routes during lithiation and delithiation processes.

## Results and discussion

**Fig. 1:**
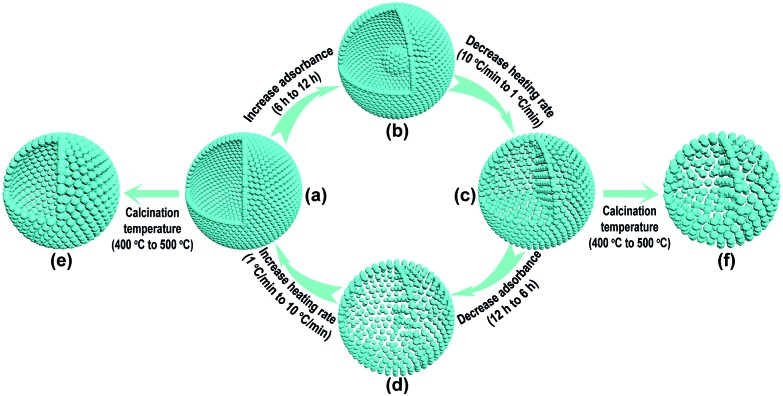
Schematic diagram of the controllable synthesis for each product. (a) TiO_2_-HNS; (b) TiO_2_-core–shell; (c) TiO_2_-PNS; (d) TiO_2_-PNS-LS; (e) TiO_2_-HNS-500 and (f) TiO_2_-PNS-500.

**Fig. 2:**
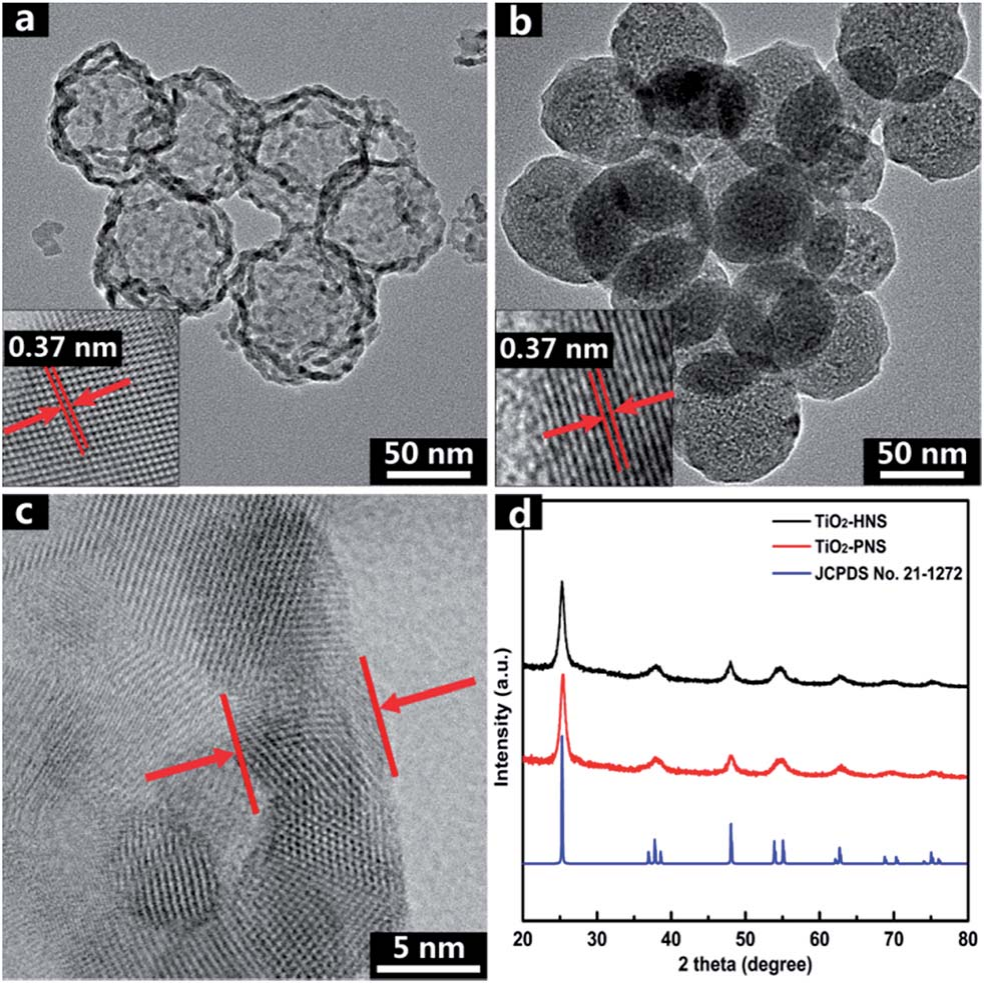
TEM micrographs of (a) TiO_2_-HNSs and (b) TiO_2_-PNSs. (c) Spherical aberration corrected TEM micrograph of the TiO_2_-HNS shell, and (d) XRD patterns of TiO_2_-HNSs and TiO_2_-PNSs.

In order to decrease the nanoparticle size in the TiO_2_-HNSs and TiO_2_-PNSs, the calcination temperature could be decreased.^37,38^ However, the micro-sized carbonaceous microspheres with diameters of around 3 μm synthesized using sucrose as a precursor could not be easily decomposed at such low temperatures after adsorbing the Ti source (Fig. S1†). Thus, much smaller quasi-nano-sized carbonaceous spheres (CNSs) with an average diameter of approximately 260 nm were synthesized *via* a hydrothermal process with glucose instead of sucrose as the precursor,^39,40^ which easily decompose at 400 °C (Fig. S1 and S2†). Based on previous work,^36,40^ a 3 M TiCl_4_ aqueous solution was used as the precursor since smaller sized Ti-coordinated cations can be formed and they are more easily adsorbed by the negatively charged carbonaceous spheres. By systematically controlling the adsorption time of the Ti source and controlling the calcination process to regulate the combustion kinetics and diffusion dynamics (see Fig. 1 and Table S1†), we are able to tune the mesostructures of the resulting products. Essentially, after leaving the CNSs in a 3 M TiCl_4_ aqueous solution for 6 h, the as-soaked CNSs were calcined at 400 °C for 4 h at a heating rate of 10 °C min^–1^ to remove the CNS templates. During the CNS removal process, the Ti source radially diffuses from the outside to the inside of the CNSs until they coalesce to form a shell. In fact, controlling the time for the precursor adsorption onto and within the CNSs significantly affected the resulting structures. In the CNSs soaked for 6 h, TiO_2_-HNSs were formed (Fig. 1a and 2a), which is ascribed to the scarce but opportune adsorbance of the Ti precursor. However, at reduced times (*i.e.*, 4 h) nearly-collapsed hollow nanospheres (Fig. S3a†) formed due to a reduced adsorbance, which was confirmed by thermogravimetric (TG) analysis (Fig. S1†). In contrast, increasing the soaking time to 12 h in a 3 M TiCl_4_ aqueous solution increased the adsorbance of TiCl_4_ onto and within the CNSs (Fig. S1†). Under these conditions, following calcination treatment at 400 °C for 4 h at a heating rate of 10 °C min^–1^, the excess Ti source remaining after forming a shell would promote the formation of a core inside the shell during the decomposition of the as-soaked CNSs, yielding TiO_2_ core–shell nanospheres (Fig. 1b and S3b†). If the calcination heating rate of the CNSs soaked for 12 h was decreased to 1 °C min^–1^, the decomposition rate of the CNSs was reduced and the Ti source would slowly shrink and aggregate continuously with the CNS templates until TiO_2_-PNSs were eventually formed (Fig. 1c and 2b). It's reasonable that the CNSs soaked for 6 h could lead to porous nanospheres with loosely aggregated nanoparticles (TiO_2_-PNS-LS) since the adsorbance of the Ti source was reduced (Fig. 1d, S1, S3c and S3d†). Moreover, TiO_2_ hollow nanospheres and porous nanospheres composed of about 15 nm sized nanoparticles (termed TiO_2_-HNS-500 and TiO_2_-PNS-500, respectively) were also prepared using the same conditions as those for the TiO_2_-HNSs and TiO_2_-PNSs, respectively, with the exception of modifying the calcination temperature to 500 °C (Fig. 1e, f, S4a and S4b†). The larger particle size is attributed to the higher calcination temperature yielding grain growth resulting in thicker shells in the TiO_2_-HNS-500 specimen compared to the TiO_2_-HNS samples.

**Fig. 3:**
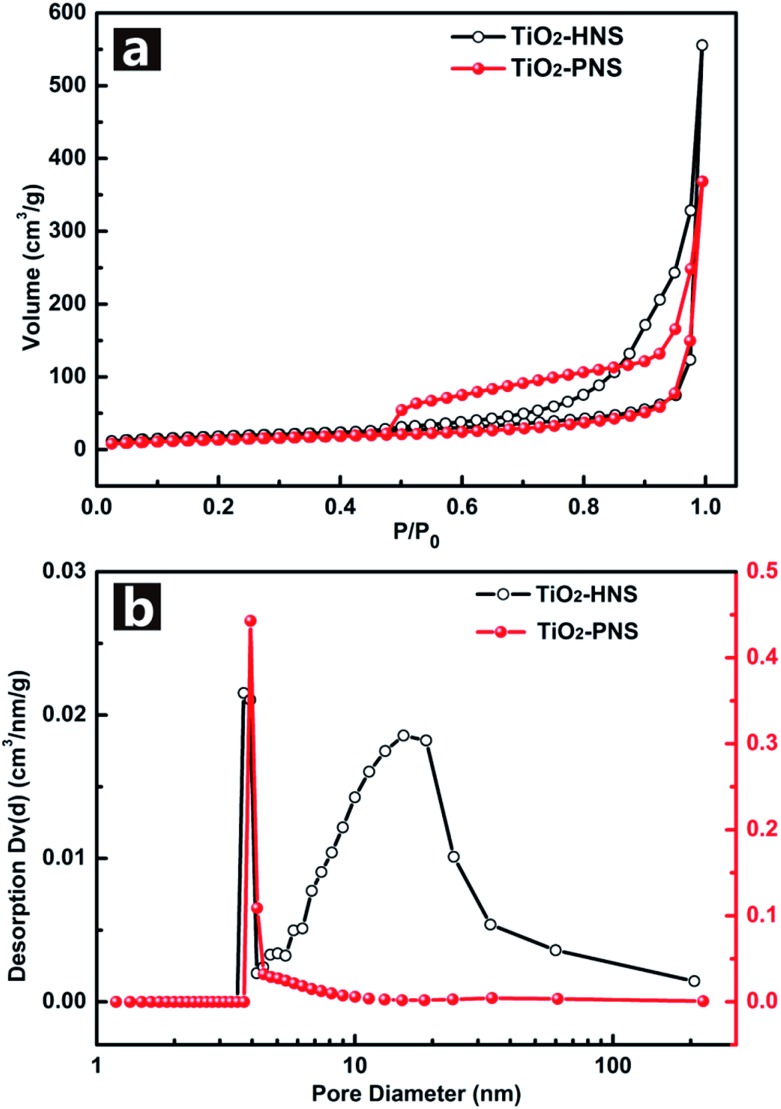
(a) Nitrogen adsorption/desorption isotherms and (b) Barret–Joyner–Halenda (BJH) pore-size distribution curves of the TiO_2_-HNSs and TiO_2_-PNSs.

The TiO_2_-HNS and TiO_2_-PNS structures are confirmed by TEM micrographs in Fig. 2a and b. Both these two kinds of nanospheres are uniform with a diameter of about 80 nm and are composed of about 7 nm sized nanoparticles (Fig. S5†). The shell of the TiO_2_-HNSs is only as thin as about 7 nm confirmed further using the spherical aberration corrected TEM images as shown in Fig. 2c, which means that the shell is composed of only a single layer of nanoparticles. The SEM micrographs in Fig. S6a and b† further show that both the TiO_2_-HNSs and the TiO_2_-PNSs are uniformly dispersed. The specific surface areas and pore size distributions are investigated using N_2_ adsorption/desorption analysis and shown in Fig. 3a and b. The TiO_2_-HNSs have a higher specific surface area of 65.8 m^2^ g^–1^ than that of 51.3 m^2^ g^–1^ for the TiO_2_-PNSs. Moreover, the size of the mesopores in the TiO_2_-PNSs is about 4 nm while the TiO_2_-HNSs have more much larger pores around 20 nm as well as a few pores around 4 nm. The effects of calcination heating rate and temperature on the mesoporous structures have also been investigated as shown in Fig. S7,† which demonstrates that a high heating rate results in large pores and a high calcination temperature would destroy the pores.

High resolution TEM (HRTEM) micrographs in the insets of Fig. 2a and b reveal the lattices of the TiO_2_-HNSs and TiO_2_-PNSs, which correspond to the {100} plane of anatase.^41^ The crystallinities of both the TiO_2_-HNSs and TiO_2_-PNSs were analyzed using X-ray powder diffraction (XRD), which confirmed the presence of crystallized anatase (JCPDS card no. 21-1272, space group: *I*41/*amd*, *a* = 3.7845 Å, *c* = 9.5143 Å) (Fig. 2d). The crystallite diameters of the TiO_2_-HNSs and TiO_2_-PNSs were calculated to be 7.3 nm and 7.1 nm, respectively, using the Scherrer formula.^42^ Raman spectra were used to further identify the composition of the TiO_2_-HNSs and TiO_2_-PNSs as shown in Fig. S8.† The Raman lines at around 144, 197 and 639 cm^–1^ can be assigned as the E_g_ modes of anatase TiO_2_. The Raman line at around 399 cm^–1^ is assigned to the B_1g_ mode of anatase and the Raman line at around 519 cm^–1^ is assigned to the A_1g_ or B_1g_ mode of anatase.^43,44^ No other Raman lines are observed, which means that the samples are pure anatase. The XRD patterns of TiO_2_-HNS-500 and TiO_2_-PNS-500 samples demonstrate that trace rutile is formed besides the main anatase phase as shown in Fig. S9† and the sizes of nanoparticles are calculated to be 16.1 nm and 15.2 nm, respectively, which is attributed to the higher calcination temperature. The small nanoparticles in the TiO_2_-HNSs and TiO_2_-PNSs are believed to be effective in the high charge/discharge current rate performance in anodes for LIBs as previously described.

**Fig. 4:**
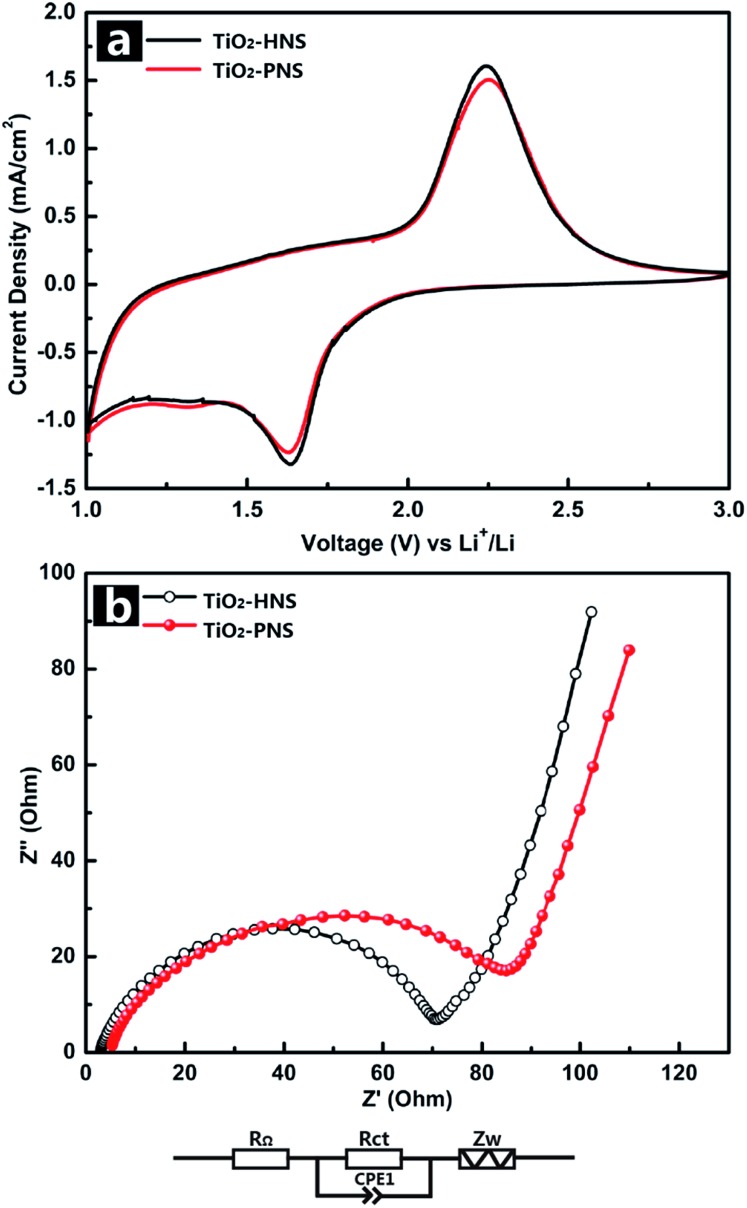
(a) Cyclic voltammetry profiles of the TiO_2_-HNSs and TiO_2_-PNSs at a scan rate of 1 mV s^–1^ between 1.0 V and 3.0 V for the first cycle, and (b) electrochemical impedance spectra (EIS) of the TiO_2_-HNSs and TiO_2_-PNSs (*R*_Ω_: external resistance, *R*_ct_: charge transfer resistance, CPE1: constant phase element, *Z*_w_: Warburg impedance).

The electrochemical behaviors of the TiO_2_-HNS and TiO_2_-PNS electrodes were measured using cyclic voltammetry (CV) at a scan rate of 1 mV s^–1^ between 1.0 V and 3.0 V (Fig. 4a). Apparent anodic and cathodic peaks are observed in both electrodes at about 2.2 V and 1.7 V, which is in agreement with the previous literature.^45^ Slight peaks below 1.5 V are also found for both the samples, which can be attributed to an irreversible capacity loss.^46^ The TiO_2_-HNSs show a higher current density than that of the TiO_2_-PNSs manifested by a higher level charge separation and electrochemical conductivity.^34^ The Nyquist plots for the samples in Fig. 4b show a single semicircle in the high frequency region corresponding to the charge-transfer resistance (*R*_ct_) and a sloping straight line in the low frequency range corresponding to solid-state diffusion of lithium (*Z*_w_).^47,48^ The *R*_ct_ of the TiO_2_-HNSs is smaller than that of the TiO_2_-PNSs, suggesting that the charge transfer for the former occurs prior to that of the latter. This may be ascribed to a higher specific surface area that enables greater contact with the electrolyte and larger pores around 20 nm (besides the 4 nm small pores), which may facilitate better electrolyte transport and strain release compared to that in the TiO_2_-PNSs (Fig. 3a, b and Table S2†). It is thus predicted that the performance of the TiO_2_-HNSs as anodes for LIBs would be enhanced.

**Fig. 5:**
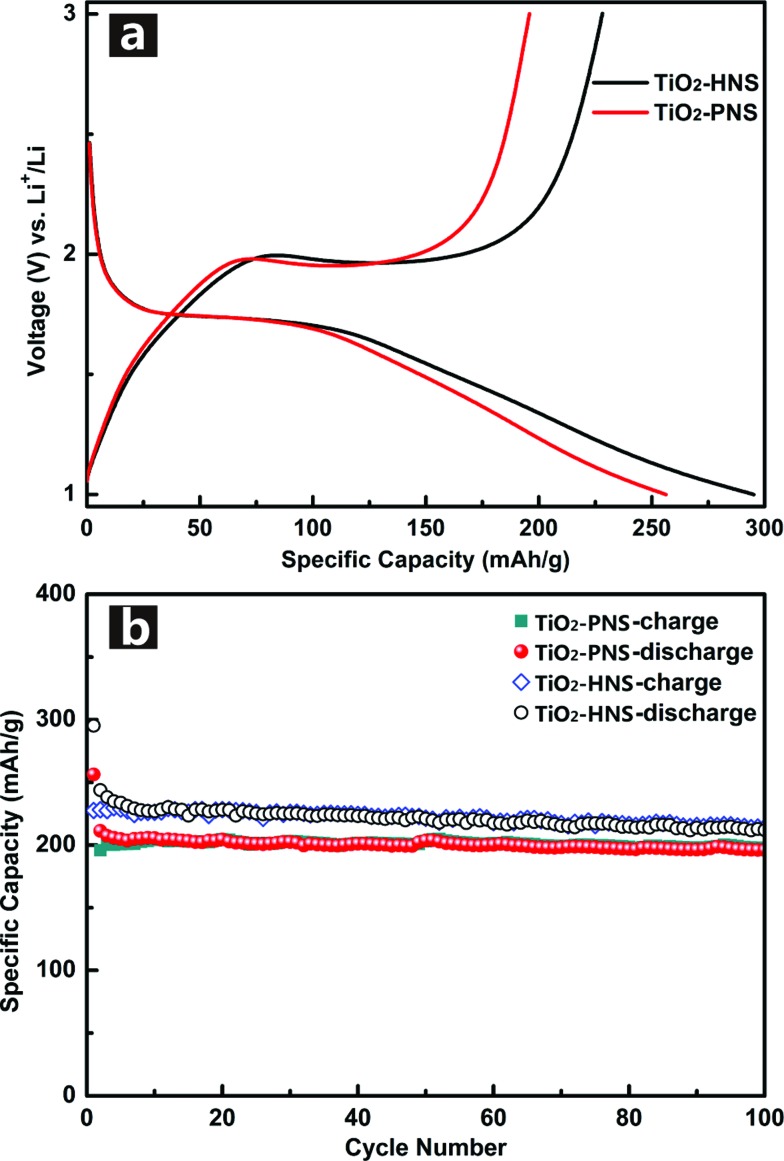
(a) Initial charge–discharge voltage profiles and (b) cycling performance of the TiO_2_-HNSs and TiO_2_-PNS at a current rate of 1 C between 1.0 and 3.0 V.

Standard TiO_2_/Li half-cells were used to measure the lithium storage properties of the TiO_2_-HNSs and TiO_2_-PNSs as anodes. Fig. 5a displays the first charge–discharge voltage profiles of the samples at a current rate of 1 C (167.5 mA g^–1^) between 1.0 V and 3.0 V. The potential falls to a plateau of 1.7 V quickly and then gradually declines to the cut-off potential of 1.0 V, consistent with previous literature.^49,50^ An initial discharge capacity of 295.2 mA h g^–1^ and charge capacity of 228.2 mA h g^–1^ are achieved for the TiO_2_-HNSs, resulting in a coulombic efficiency of 77.3%. The TiO_2_-PNSs exhibit an initial discharge capacity of 256.3 mA h g^–1^ and charge capacity of 195.9 mA h g^–1^, leading to a coulombic efficiency of 76.4%. The slightly lower coulombic efficiency is ascribed to the irreversible capacity loss, corroborating the CV measurement. Fig. 5b shows the cycling performance of the TiO_2_-HNSs and TiO_2_-PNSs at a current rate of 1 C between 1.0 V and 3.0 V for 100 cycles. After 100 cycles, the TiO_2_-HNSs show a higher capacity than the TiO_2_-PNSs (211.9 mA h g^–1^ *vs.* 196.0 mA h g^–1^). The higher capacity of the TiO_2_-HNSs is ascribed to the higher specific surface area, which can lead to more surface lithium storage^51^ (Table S2†). Although demonstrating a slightly lower capacity, the TiO_2_-PNSs show a capacity retention of 92.8% with respect to the reversible specific capacities after 100 cycles, which is higher than that of the TiO_2_-HNSs (86.9%). This results from the very thin shell of the TiO_2_-HNSs, which is composed of only a single layer of nanoparticles and can fracture due to volume expansion during the charge and discharge processes (as seen in the TEM micrographs after 100 cycles, Fig. S10†). However, the TiO_2_-PNSs still maintain an unchanged stable structure leading to outstanding cycling performance, significantly better than that of commercial TiO_2_ Degussa P25.^36^ Moreover, the mass energy density of the TiO_2_-PNSs would be higher because of their more effective volumetric occupation compared to the TiO_2_-HNSs.

**Fig. 6:**
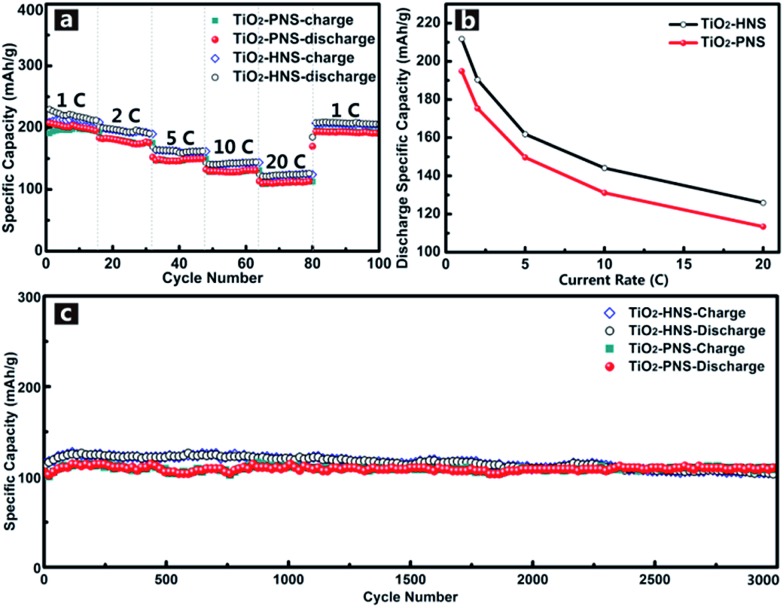
(a) Cycling performance at various charge–discharge current rates of the TiO_2_-HNSs and TiO_2_-PNSs between 1.0 and 3.0 V. (b) Statistics of the discharge specific capacities at various current rates according to the rate performance in (a). (Each discharge capacity summarized here is the last cycle of each current rate.) (c) Long-term cycling performance of the TiO_2_-HNSs and TiO_2_-PNSs at a high current rate of 20 C for 3000 cycles.

Considering the importance of the potential for high rate applications, the cycling performance of the TiO_2_-HNSs and TiO_2_-PNSs at various charge and discharge current rates was measured. As shown in Fig. 6a, the capacity drops slowly as the current rate is increased up to 20 C (approximately 3 min for a charge or discharge process) from 1 C. A capacity of 125.9 mA h g^–1^ for the TiO_2_-HNSs and 113.4 mA h g^–1^ for the TiO_2_-PNSs can be achieved even at a high current rate of 20 C. A high discharge capacity of 205.4 mA h g^–1^ for the TiO_2_-HNSs and 190.1 mA h g^–1^ for the TiO_2_-PNSs can still be achieved when the current rate is reduced back to 1 C. The high discharge capacities at various current rates are summarized in Table S3† and are presented in Fig. 6b. This superior rate performance can be attributed to the special stable structures and fine particle sizes, which lead to very short lithium ion diffusion lengths and electron pathways during the lithium insertion and expulsion.^30,31,52^

In comparison, the rate performance of the TiO_2_-HNS-500 and TiO_2_-PNS-500 samples composed of larger nanoparticles was also measured as shown in Fig. S11† and the data are summarized in Table S3.† At a current rate of 1 C, the TiO_2_-HNS-500 sample shows a discharge capacity of 172.5 mA h g^–1^ while the TiO_2_-PNS-500 sample shows a discharge capacity of 145.7 mA h g^–1^. Discharge capacities of 65.4 and 54.7 mA h g^–1^ for the TiO_2_-HNS-500 and TiO_2_-PNS-500 samples, respectively, are achieved at a high current rate of 20 C. The capacities at 20 C are only 37.91% and 37.54% of the capacities at 1 C for the TiO_2_-HNS-500 and TiO_2_-PNS-500 samples, respectively, which are much lower than the results of 59.47% and 58.20% for the TiO_2_-HNSs and TiO_2_-PNSs, respectively. This results from the smaller nanoparticles in the TiO_2_-HNS and TiO_2_-PNS specimens, which could decrease the lithium ion diffusion lengths and shorten the electron pathways during the charge and discharge processes.^52^

The long-term cycling performance of the TiO_2_-HNSs and TiO_2_-PNSs at a high current rate of 20 C has also been demonstrated (Fig. 6c). Even after 3000 cycles, the TiO_2_-HNSs and TiO_2_-PNSs still maintain high discharge capacities of 103.0 mA h g^–1^ and 110.2 mA h g^–1^, respectively, with high retentions of 80.97% and 95.2% compared to the highest discharge capacities around the 116^th^ cycle, respectively. The TiO_2_-PNSs show a significantly lower capacity loss because of their more stable structure than that of the TiO_2_-HNSs. The superior long-term cycling performance endows these nanospheres with great potential for application.

## Conclusions

In summary, uniform TiO_2_ hollow and mesoporous nanospheres composed of ultrasmall nanoparticles were successfully fabricated using quasi-nano-sized carbonaceous spheres with a diameter of 260 nm as templates. The hollow and mesoporous structures could be controlled by controlling the heating rate of the calcination process and the adsorbance of TiCl_4_. When measured as anodes for LIBs, both TiO_2_ hollow and mesoporous nanospheres show good cycling performance, high specific capacity and high current rate performance. High specific capacities of 211.9 and 196.0 mA h g^–1^ are achieved for TiO_2_ hollow and mesoporous nanospheres at a current rate of 1 C after 100 cycles. Even at a high current rate of 20 C, they can still retain high specific capacities of 125.9 and 113.4 mA h g^–1^. TiO_2_ hollow and mesoporous nanospheres demonstrate long-term discharge capacities of 103.0 and 110.2 mA h g^–1^ after 3000 cycles at 20 C. The superior performance especially at high current rates is ascribed to the stable porous structures composed of ultrasmall nanoparticles, which enhance the interfacial contact area with the electrolyte and contribute to much shorter lithium ion diffusion lengths and electron transfer pathways. These TiO_2_ nanospheres have a great potential to be used for safe and high charge/discharge current rate devices.
