# Supplementary material for: Controllable synthesis of mesostructures from TiO2 hollow to porous nanospheres with superior rate performance for lithium ion batteries
Source: Chem Sci. 2015 Oct 26;7(1):793–8. doi: 10.1039/c5sc03203b (PMC5580042; doi:10.1039/c5sc03203b)
Supplement: Supplementary file 1 [file SC-007-C5SC03203B-s001.pdf]

Electronic Supplementary Material (ESI) for Chemical Science.
This journal is © The Royal Society of Chemistry 2015

Controllable Synthesis of Mesostructures – from TiO_2 Hollow to Porous Nanospheres with Superior Rate Performance for Lithium Ion Batteries

Hao Ren,[a] Jiajia Sun,[a] Ranbo Yu,[a,*] Mei Yang,[b] Lin Gu,[c] Porun Liu,[d] Huijun Zhao,[d] David Kisailus,[e] and Dan Wang[b,d]

[a] Department of Physical Chemistry, School of Metallurgical and Ecological Engineering, University of Science & Technology Beijing

No. 30, Xueyuan Road, Haidian District, Beijing 100083, P. R. China.

[b] National Key Laboratory of Biochemical Engineering, Institute of Process Engineering, Chinese Academy of Sciences

No. 1, Bei Er Tiao, Zhongguancun, Beijing 100190, P. R. China.

[c] Laboratory for Advanced Materials & electron Microscopy, Beijing National Laboratory for Condensed Matter Physics, Institute of Physics, Chinese Academy of Sciences, Beijing 100190, P. R. China.

[d] Centre for Clean Environment and Energy, Gold Coast Campus, Griffith University, Southport, Queensland 4222, Australia.

[e] Department of Chemical and Environmental Engineering, University of California, Riverside, CA 92521.

Supporting Information

Experimental Section

Materials: All reagents purchased from Beijing Chemical Reagent Factory were of analytical grade and used without further purification.

Synthesis of quasi-nano-sized carbonaceous spheres: The carbonaceous spheres were

prepared by a hydrothermal method. Typically, 89 g of glucose was dissolved in 250 mL of deionized water to form a uniform solution and sealed into a 500 mL Teflon reactor. The reactor was then put into an oven for 5.5 h at 180 °C and cooled to room temperature. The brown product was centrifuged at 9000 rpm for 8 min and washed 3 times with deionized water and ethanol followed by drying at 80 °C overnight. These quasi-nano-sized carbonaceous spheres (CNS) were used as templates.

Synthesis of hollow TiO_2 nanospheres (TiO_2-HNS) and mesoporous TiO_2 nanospheres (TiO_2-PNS): To synthesize TiO_2, a 3 M $TiCl_4$ aqueous precursor solution was made by slowly adding 17 mL of pure $TiCl_4$ into a mixture of ice and deionized water to form a 50 mL solution. 1 g of the as-prepared quasi-nano-sized carbonaceous spheres were added into the $TiCl_4$ aqueous solution and ultrasonicated for 15 min to disperse the CNS followed by aging for 6 h (12 h for mesoporous nanospheres) under stirring. The products were centrifuged at 9000 rpm for 8 min and washed once with acetic acid and dried at 80 °C overnight. The products were subsequently calcined at 10 °C min^{-1} (1 °C min^{-1} for porous nanospheres) to 400 °C for 4 h resulting in white TiO_2-HNS and TiO_2-PNS products. For TiO_2-HNS-500 and TiO_2-PNS-500, the calcination temperature was 500 ^{o}C. For TiO_2-HNS-600 and TiO_2-PNS-600, the calcination temperature was 600 ^{o}C.

TiO_2 core-shell nanospheres were formed when the 12 h adsorbed CNS was calcined at a heating rate of 10 ^{o}C min^{-1} to 400 °C for 4 h while porous nanospheres with loose aggregated nanoparticles (TiO_2-PNS-LS) were formed when the 6 h adsorbed CNS was calcined at a heating rate of 1 ^{o}C min^{-1} to 400 °C for 4 h.

The detailed synthesis conditions of different types of TiO_2 nanospheres have been summarized in **Table S1**.

Materials characterization: Scanning electron microscopy (SEM) images were obtained using a JSM-6700 microscope operated at 5.0 kV. Transmission electron microscope (TEM) images were taken using a FEI Tecnai F20 instrument operated at an accelerating voltage of 200 kV. Thermogravimetric (TG) analyses were performed on a TGA Q500 thermogravimetric analyzer in air with a heating rate of 10°C min^{-1}. Powder X-ray diffraction (XRD) patterns were recorded on a Panalytical X'Pert PRO MPD [Cu Kα radiation (λ = 1.5405 Å)], operating at 40 kV and 30 mA. Raman spectra were measured with a HORIBA Raman system model LabRAM HR Evolution spectrometer. The 532 nm line of argon ion laser was used as the excitation source and the resolution of the Raman instrument is ca. 0.65 cm^{-1}. The Raman band of the silicon wafer at 520.7 cm^{-1} was used to calibrate the spectrometer. The nitrogen adsorption-desorption isotherms were measured at -196°C using a Quantachrome Autosorb-1MP sorption analyzer. Samples were degassed under vacuum at 200°C overnight prior to analysis.

Electrochemical measurements: Electrochemical measurements of the prepared TiO_2 hollow and porous nanosphere samples were carried out using CR2032 coin type cells with lithium metal as the counter and reference electrodes at room temperature. The electrolyte was 1 M $LiPF_6$ in a 50:50 w/w mixture of ethylene carbonate and diethyl carbonate. The working electrode was fabricated by compressing a mixture of the active materials, conductive material (carbon black), and binder (polyvinylidene

fluoride) in a weight ratio of TiO_2/carbon/PVDF = 7:2:1 onto a copper foil current collector. The typical mass loading of active materials for each coin cell was approximately 2 mg. The cells were assembled in an argon-filled glove box with the concentrations of moisture and oxygen below 0.1 ppm. The electrode capacity was measured by a galvanostatic discharge-charge method between 1.0 V and 3.0 V at 1 C, 2 C, 5 C and 10 C and 20 C with a Neware BST-5 V 5 mA battery test system.

Samples were examined using cyclic voltammetry from 1.0-3.0 V (vs. Li^+/Li) at a constant scan rate of 1 mV s^{-1}. Electrochemical impedance spectra (EIS) tests were carried out over a frequency range of 0.1 to 10^5 Hz with an AC amplified voltage of 5 mV.

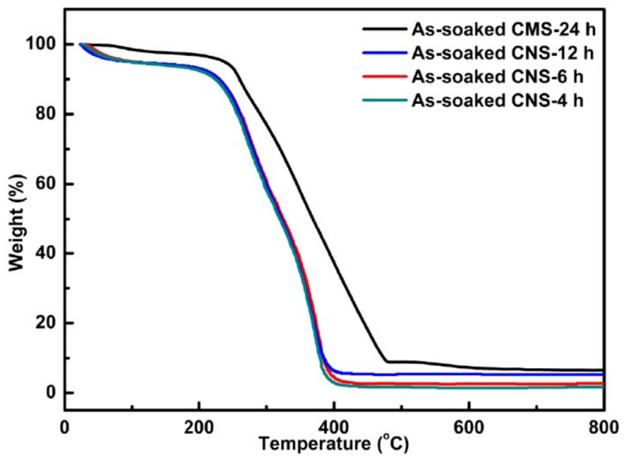

Figure S1. Thermogravimetric heating curves of the as-adsorbed carbonaceous microspheres (CMS) for 24 h and as-adsorbed quasi-nano-sized carbonaceous nanospheres (CNS) for 4h, 6h and 12 h in a 3 M $TiCl_4$ aqueous solution.

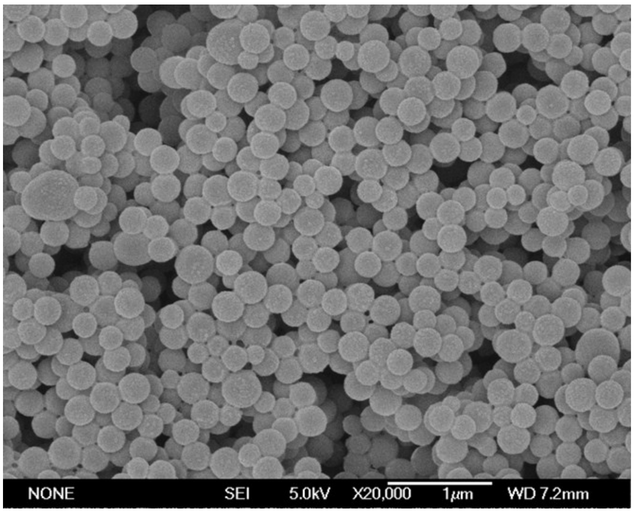

Figure S2. SEM micrograph of as-prepared CNS.

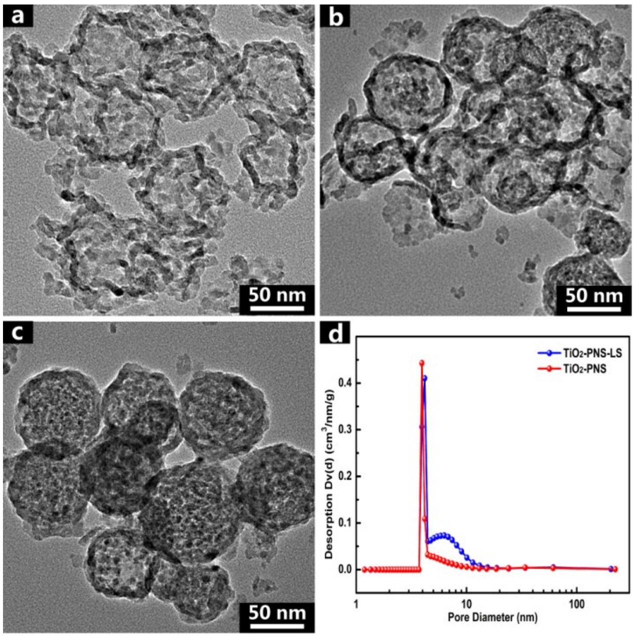

Figure S3. TEM micrographs of samples calcined at 400 ^{o}C for 4h (a) at a heating rate of 10 ^{o}C min^{-1} after adsorbing a 3 M $TiCl_4$ aqueous solution for 4 h (collapsed TiO_2-HNS); (b) at a heating rate of 10 ^{o}C min^{-1} after adsorbing a 3 M $TiCl_4$ aqueous solution for 12 h (TiO_2-core-shell nanospheres); (c) at a heating rate of 1 ^{o}C min^{-1} after adsorbing a 3 M $TiCl_4$ aqueous solution for 6 h (TiO_2-PNS-LS); (d) Barret-Joyner-Halenda (BJH) pore-size distribution curves of TiO_2-PNS and TiO_2-PNS-LS.

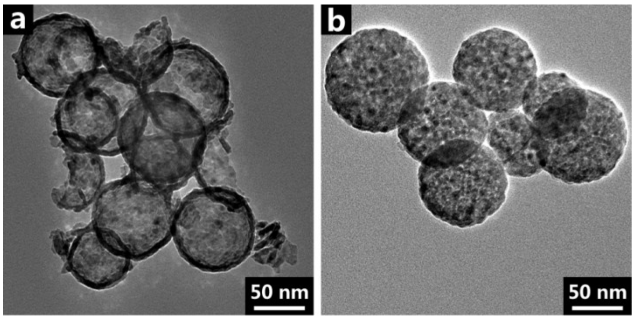

Figure S4. TEM micrographs of (a) TiO_2-HNS-500 which were calcined at 500 ^oC for 4 h at a heating rate of 10 ^oC min^{-1} after absorbing 3 M $TiCl_4$ aqueous solution for 6 h and (b) TiO_2-PNS-500 which were calcined at 500 ^oC for 4 h at a heating rate of 1 ^oC min^{-1} after absorbing 3 M $TiCl_4$ aqueous solution for 12 h.

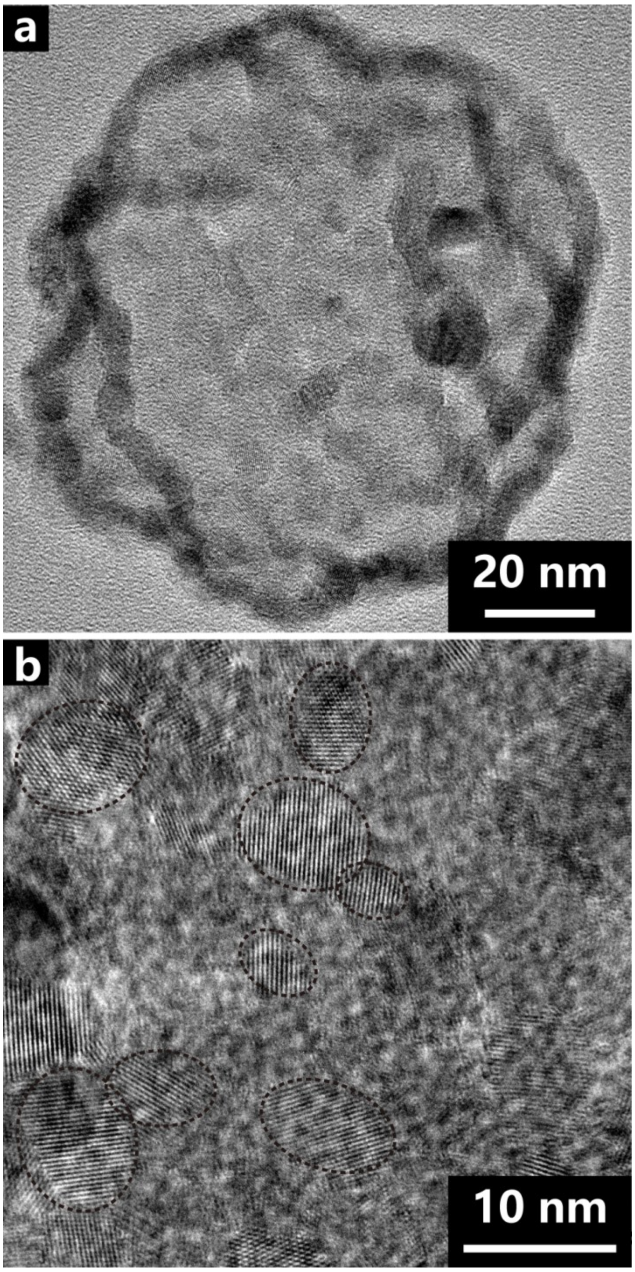

Figure S5. TEM images of TiO_2-HNS highlighting nanoparticles within the shell.

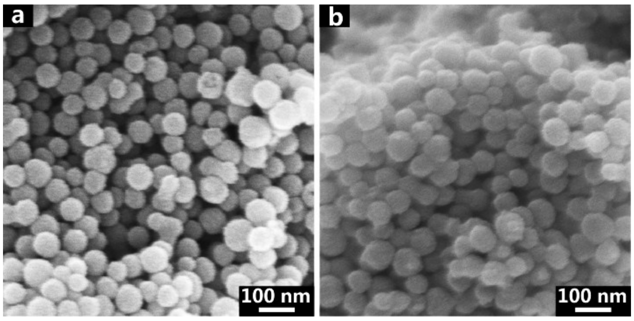

Figure S6. SEM micrographs of (a) TiO_2-HNS and (b) TiO_2-PNS.

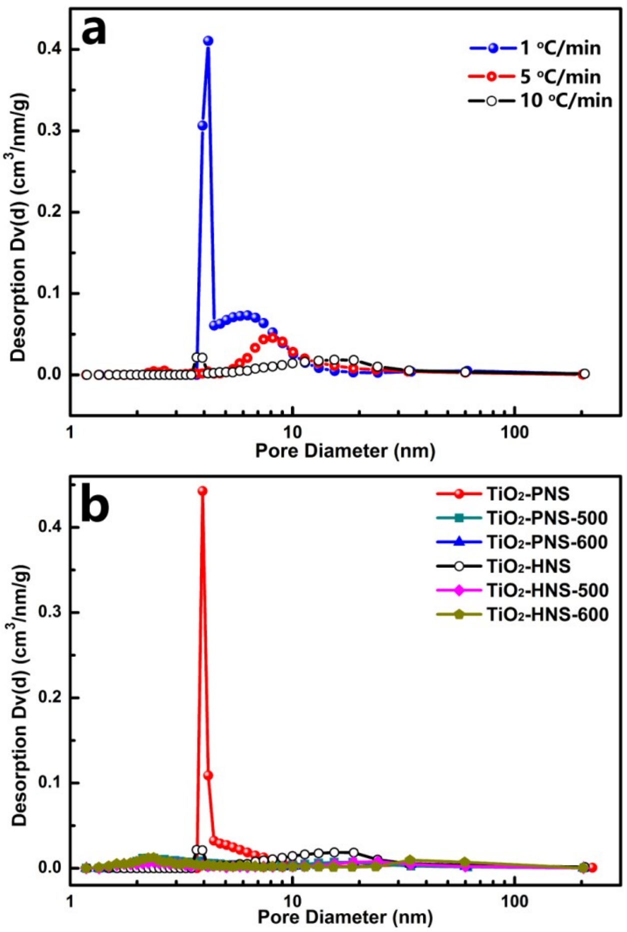

Figure S7. (a) Pore size distribution of TiO_2 nanospheres obtained by calcination at 400 °C for 4 h with a heating rate of 1, 5 and 10 °C/min after adsorbing a 3 M $TiCl_4$ aqueous solution for 6 h; (b) Pore size distribution of TiO_2 porous nanospheres calcinated under 400, 500 and 600 °C for 4 h at a heating rate of 1 °C/min after adsorbing a 3 M $TiCl_4$ aqueous solution for 12 h (TiO_2-PNS, TiO_2-PNS-500 and TiO_2-PNS-600), and TiO_2 hollow nanospheres calcinated under 400, 500 and 600 °C for 4 h at a heating rate of 10 °C/min after adsorbing a 3 M $TiCl_4$ aqueous solution for 6 h (TiO_2-HNS, TiO_2-HNS-500 and TiO_2-HNS-600).

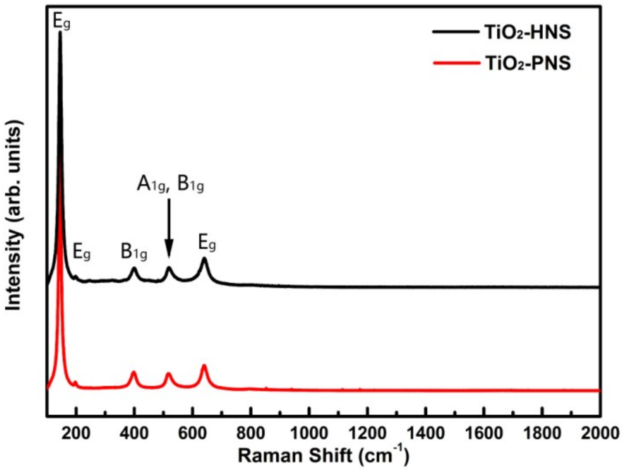

Figure S8. Raman spectra of TiO_2-HNS and TiO_2-PNS.

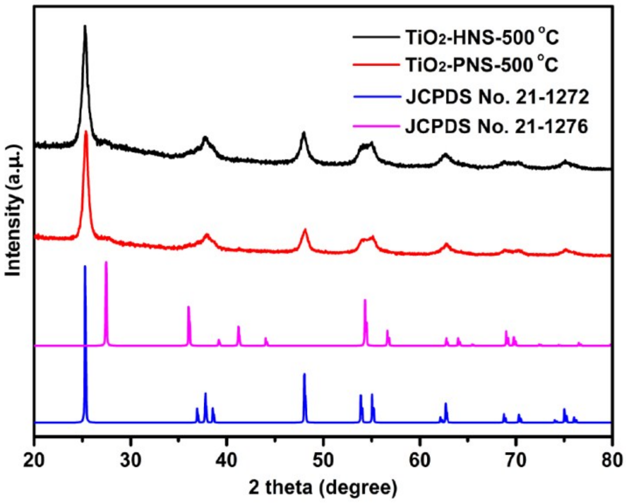

Figure S9. XRD patterns of TiO_2-HNS-500 and TiO_2-PNS-500.

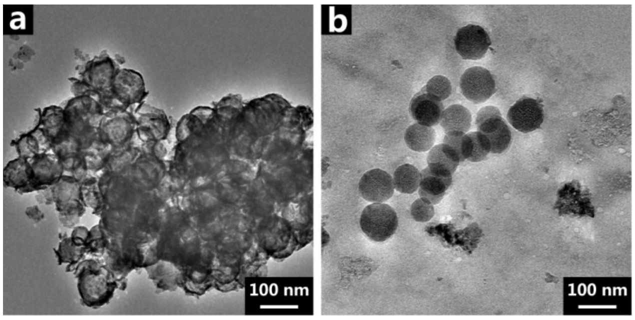

Figure S10. TEM micrographs of (a) TiO_2-HNS and (b) TiO_2-PNS after 100 cycles at a current rate of 1 C.

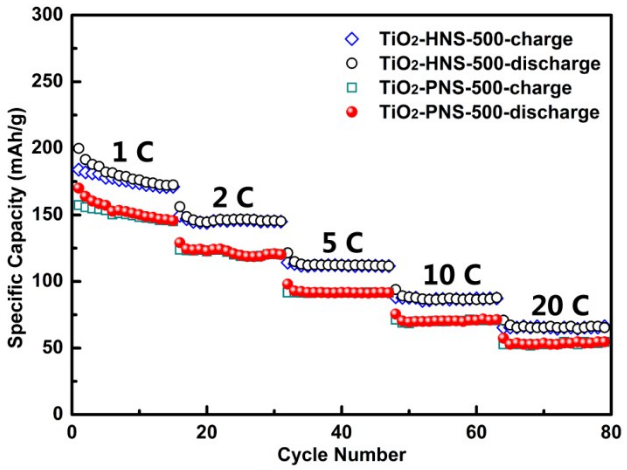

Figure S11. Cycling performance of TiO_2-HNS-500 and TiO_2-PNS-500 at various charge-discharge current rates from 1 C to 20 C between 1.0 and 3.0 V.

Table S1. Synthesis conditions of the as-prepared TiO_2 nanospheres.

Sample	Adsorbing Time	Calcination Heating Rate	Calcination Temperature	Label Corresponding to Figure 1
TiO_2-HNS	6 h	10 °C/min	400 °C for 4 h	(a)
TiO_2-core-shell	12 h	10 °C/min	400 °C for 4 h	(b)
TiO_2-PNS	12 h	1 °C/min	400 °C for 4 h	(c)
TiO_2-PNS-LS	6 h	1 °C/min	400 °C for 4 h	(d)
TiO_2-HNS-500	6 h	10 °C/min	500 °C for 4 h	(e)
TiO_2-PNS-500	12 h	1 °C/min	500 °C for 4 h	(f)

Table S2. Summary of parameters from nitrogen adsorption-desorption isotherms of the as-prepared TiO_2 nanospheres.

Sample	Specific Surface Area (m^2/g)	Main Pore Size (nm)	Pore Volume (cm^3/g)
TiO_2-HNS	65.8	4 / 20	0.86
TiO_2-core-shell	65.6	4 / 20	0.82
TiO_2-PNS	51.3	4	0.57

TiO_2-PNS-LS	60.5	4 / 6	0.66

Table S3. Summary of discharge capacities of the as-prepared TiO_2-HNS, TiO_2-PNS, TiO_2-HNS-500 and TiO_2-PNS-500 at various current rates between 1.0 and 3.0 V. (Each discharge capacity summarized here is the last cycle of each current rate.)

Samples	1 C (mAh/g)	2 C (mAh/g)	5 C (mAh/g)	10 C (mAh/g)	20 C (mAh/g)
TiO_2-HNS	211.7	190.3	161.8	144.1	125.9
TiO_2-PNS	194.8	175.3	149.7	131.1	113.4
TiO_2-HNS-500	172.5	145.3	111.7	87.8	65.4
TiO_2-PNS-500	145.7	120.2	91.5	71.4	54.7
